# Soil Nutrient Estimation and Mapping in Farmland Based on UAV Imaging Spectrometry

**DOI:** 10.3390/s21113919

**Published:** 2021-06-06

**Authors:** Xiaoyu Yang, Nisha Bao, Wenwen Li, Shanjun Liu, Yanhua Fu, Yachun Mao

**Affiliations:** 1 College of Resources and Civil Engineering, Northeastern University, Shenyang 110819, China; yangxiaoyu368@163.com (X.Y.); liusjdr@126.com (S.L.); maoyachun@mail.neu.edu.cn (Y.M.); 2 School of Geographical Sciences and Urban Planning, Arizona State University, Tempe, AZ 85287, USA; wenwen@asu.edu; 3 JangHo Architecture College, Northeastern University, Shenyang 110169, China; fuyanhua@mail.neu.edu.cn

**Keywords:** unmanned aerial vehicle, hyperspectral image, extreme learning machine, soil nutrient estimation, feature selection

## Abstract

Soil nutrient is one of the most important properties for improving farmland quality and product. Imaging spectrometry has the potential for rapid acquisition and real-time monitoring of soil characteristics. This study aims to explore the preprocessing and modeling methods of hyperspectral images obtained from an unmanned aerial vehicle (UAV) platform for estimating the soil organic matter (SOM) and soil total nitrogen (STN) in farmland. The results showed that: (1) Multiplicative Scattering Correction (MSC) performed better in reducing image scattering noise than Standard Normal Variate (SNV) transformation or spectral derivatives, and it yielded a result with higher correlation and lower signal-to-noise ratio; (2) The proposed feature selection method combining Successive Projections Algorithm (SPA) and Competitive Adaptive Reweighted Sampling algorithm (CARS), could provide selective preference for hyperspectral bands. Exploiting this method, 24 and 22 feature bands were selected for SOM and STN estimation, respectively; (3) The particle swarm optimization (PSO) algorithm was employed to obtain optimized input weights and bias values of the extreme learning machine (ELM) model for more accurate prediction of SOM and STN. The improved PSO-ELM model based on the selected preference bands achieved higher prediction accuracy (R^2^ of 0.73 and RPD of 1.91 for SOM, R^2^ of 0.63, and RPD of 1.53 for STN) than support vector machine (SVM), partial least squares regression (PLSR), and the ELM model. This study provides an important guideline for monitoring soil nutrient for precision agriculture with imaging spectrometry.

## 1. Introduction

Soil organic matter (SOM) and soil total nitrogen (STN) are two important variables that reflect soil quality and soil fertility [1], as they can improve the physical, chemical, and biological properties of soil and provide humic acids and carbon sources for plant growth [2,3]. The geographical distribution of SOM and STN is useful information for crop growers. Therefore, accurate and timely monitoring of their geographical distribution is essential for farmland management in precision agriculture [4].

Due to the presence of functional groups such as C-H, -COOH, -OH, and N-H in soil organic compounds corresponding to the electromagnetic radiation response, there are obvious spectral characteristics in the visible and near-infrared (VIS-NIR) regions. This makes proximal reflectance spectrometry and imaging spectrometry using VIS-NIR beneficial for the quantification of soil properties. Currently, spectrometry in the laboratory has been widely applied to the quantitative inversion of SOM based on the organic matter sensitive bands that exist in the visible range of 550~770 nm and the near-infrared range of 1300~1500 nm [5,6]. A significant correlation was also found between soil spectra and nitrogen content at 1880~1890 nm and 510 nm [7].

However, the spectral characteristic and analysis in the laboratory on collected samples cannot provide a spatially continuous distribution of soil properties in a specific area [8]. Hyperspectral imaging spectrometry has the advantages of capturing rich spectral and spatial information as well as surface information [9]. Hyperspectral remote sensing exploits hyperspectral sensors from satellites, airplanes, and unmanned aerial vehicles (UAVs) to monitor the Earth’s surface. The satellite hyperspectral sensors, such as EO-1 Hyperion can be exploited to predict soil organic carbon with a root mean square error (RMSE) of 0.73 at the regional scale [10]. However, EO-1 Hyperion data are only available before 2014, which limits the studies and applications of soil properties estimation in recent years. The GF-5 satellite with a spatial resolution of 30 m and 330 spectral bands was launched in 2018, and the data collected by this satellite have been used in soil estimation and monitoring. Meng et al. developed a regional-scale soil organic carbon prediction model (R^2^ = 0.79, RPD = 1.46) for random forest using the spectral indices filtered from the hyperspectral data of GF-5 AHSI [11]. Generally, satellite data with moderate spatial resolution are suitable for large regional-scale estimation. As for field or small-scale monitoring, the prediction accuracy of soil properties cannot meet the requirements of precision agriculture management due to mixed pixel issues. The low temporal resolution of satellite-based hyperspectral imagery also limits its use in real-time monitoring of precision agriculture.

Airborne hyperspectral imagery provides data with a high spectral and spatial resolution that may be useful to map culture land which has different types of soils and crops over a large range [12]. Hbirkou et al. [13] predicted SOC over a small-scale bare and fine agricultural soil using the data obtained from aircraft-mounted hyperspectral sensor HyMap and achieved prediction accuracy of RMSEP = 0.76 g kg^−1^ and RPD = 2.08. The aircraft typically maintain an operation height between 1000 to 5000 m above ground to acquire high-resolution images (1 m~5 m resolution). Weather conditions and ground exposure are the primary factors that limit the use of hyperspectral imagers. The aircraft must fly when the sky is clear and there is an adequate exposure of surface soil [12]. Besides, there is often a long planning time for flight missions that are often associated with high operation cost [14].

Compared with aerial systems, the UAVs fly at lower altitudes and are not subject to air traffic control [15], and they have more advantages in practical applications because of their lower cost and higher operational flexibility, which is fundamental for precise agriculture monitoring [16]. UAV-based multispectral imaging is mostly applied to classification and mapping [17], but it fails to meet the needs of quantitative soil estimation due to the limitation of spectral resolution [18]. UAV-based hyperspectral imaging provides an alternative for detecting fine-scale soil properties. Hu et al. [19] demonstrated that the UAV hyperspectral system is effective and crucial for field-scale soil feature monitoring and mapping.

In addition, it is a very challenging research topic for collecting, processing, and analyzing hyperspectral images because of the embedded imagery noise, massive data volume, high dimensionality, and complexity in the data content [17,20,21]. Machine learning techniques, such as support vector machine (SVM), random forest (RF), and extreme learning machine (ELM) have been exploited to analyze hyperspectral features and investigate various soil characteristics. Partial least squares regression (PLSR) is a regression model that has been widely used for soil properties prediction based on hyperspectral features [22]. SVM is a powerful tool to analyze hyperspectral data because of its capability to very efficiently process multiple variables and solve non-linear problems [23]. Honkavaara et al. [24] applied SVM on hyperspectral imagery collected by UAV to predict crop biomass. ELM is a machine learning algorithm that is frequently used to train feed-forward neural networks which have a single hidden layer. The weights in the network is learned through solving a Moore–Penrose (MP) generalized inverse linear problem [25]. Compared with the traditional backpropagation neural network, ELM can provide a higher calculation speed and better generalization performance, as well as avoid issues in the gradient-based training methods such as difficulties in determining a stopping criteria and a proper learning rate [26]. Ge et al. [27] concluded that the combination of preprocessed spectral indices and ELM algorithm allowed an estimation of soil moisture content with a high accuracy (R^2^_val_ = 0.907) using hyperspectral imagery from UAV. Huang et al. [28] demonstrated that ELM tended to achieve better performance in terms of scalability and model generalization and it also yielded a much faster learning speed than support vector machines. However, more hidden neurons may lead ELM to converge slowly [29]. You et al. [30] exploited the use of the particle swarm optimization (PSO) for input weights selection to help ELM with fewer hidden neurons achieve good generalization performance. Compared to other optimization strategies, PSO is easy to implement and has a smaller parameter space [31].

This study focused on improving SOM and STN prediction and mapping accuracy through UAV hyperspectral imagery denoising, preference bands selection, and model optimization. This work aimed to (1) explore the optimal denoising method for UAV hyperspectral imagery by comparing the methods including Multiplicative Scattering Correction (MSC), Standard Normal Variate transformation (SNV), the first derivate (FD), and the second derivate (SD); (2) propose a hyperspectral bands selection method by combining Successive Projections Algorithm (SPA) and Competitive Adaptive Reweighted Sampling algorithm (CARS); (3) establish an accurate SOM prediction model by comparing the performance of traditional methods and improved extreme learning machine algorithm.

## 2. Data and Methods

### 2.1. Study Site and Field Sampling

**Figure 1:**
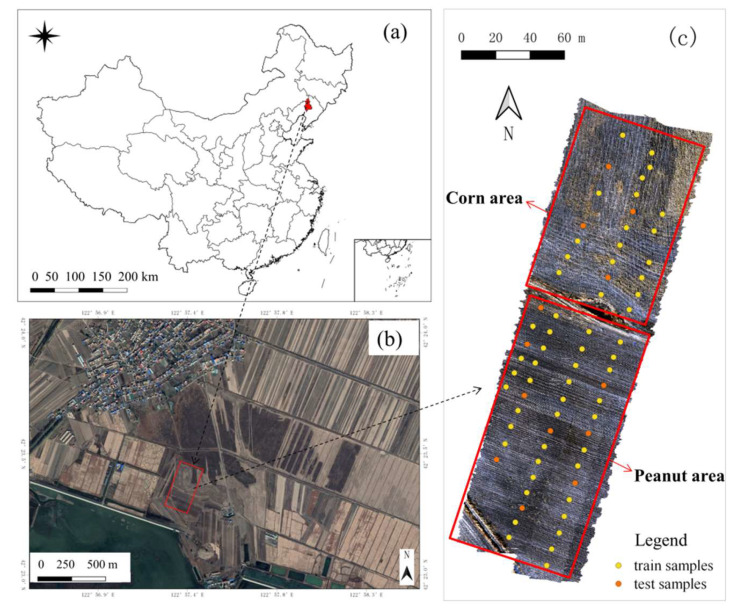
Geographical location of the study site in China (**a**), its surrounding environment (**b**), and the soil samples distribution (**c**).

The site (42°23′ N and 122°57′ E) chosen for this study was located in the north of Shenyang, Liaoning Province, Northeast China (Figure 1). The area belongs to the Songliao Plain, and it is mainly used as continuous agricultural fields as shown in Figure 1b. The area is predominantly flat with an altitude of approximately 60 m and has a continental monsoon climate in the North Temperate Zone. The average annual temperature of this area is 6.7 °C, and the average annual precipitation is 600 mm. According to the Genetic Soil Classification of China [32], this area’s soil types are mainly brown soil (Luvisols) and meadow soils (Cambisols), which are fertile, especially in terms of organic matter and nitrogen [33]. Crops planted in this area are mainly corn and peanut.

The soil samples were collected following the grid sampling method, and 68 sampling cells (1 m × 1 m) were collected uniformly at 0~10 cm depth of the study soil in November 2019 after the crops were harvested. From November to April, the farmland is idle with no vegetation cover due to the cold weather, which is a period with good conditions for satellite and airborne image acquisition. A hand-held global positioning system (GPS) was used to record the geographic coordinates at each sampling site [34]. The soil samples were ground, air-dried, and sieved to a size less or equal to 0.25 mm. Finally, the content of SOM and STN was measured through the K_2_Cr_2_O_7_–H_2_SO_4_ oxidation method and the modified Kjeldahl method, respectively [35].

**Figure 2:**
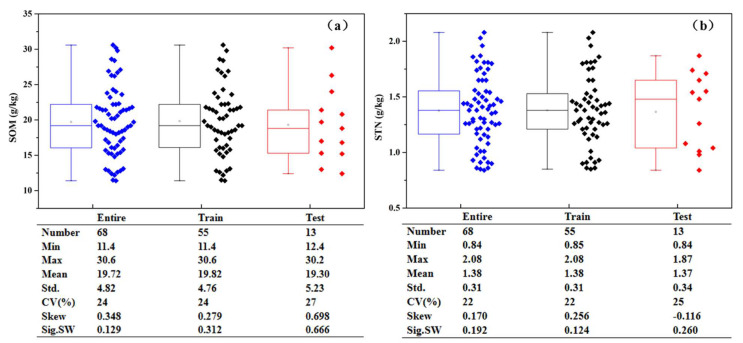
The statistical results of SOM (**a**) and STN (**b**) for the entire, training, and test datasets. CV indicates coefficients of variation (%), Skew indicates skewness, and Sig. SW indicates the significance of the Shapiro–Wilk normality test.

As shown in Figure 2a, SOM content fell within a range of 11.4~30.6 g kg^−1^, with a mean value of 19.72 g kg^−1^. The coefficient of variation (CV)—the ratio between standard deviation and the mean value, was 24%. The Shapiro–Wilk (S–W) test confirmed that the SOM content followed a normal distribution. Meanwhile, as shown in Figure 2b, STN content fell within a range of 0.84~2.08 g kg^−1^, with a mean value of 1.38 g kg^−1^. The CV of STN content was 22%, and the S–W test confirmed that the STN content followed a normal distribution. The samples were split into 80% for training and 20% for test [36]. Fifty-five train samples and 13 test samples were selected using stratified random sampling based on the content of SOM and STN, then they were fine-tuned according to the statistical and spatial distribution of the samples, which makes the test samples follow a normal distribution and spatially representative as illustrated in Figure 1c. The statistical distribution of the training set and the test set is similar to the entire sample set of SOM and STN.

### 2.2. UAV Data Acquisition and Preprocessing

**Figure 3:**
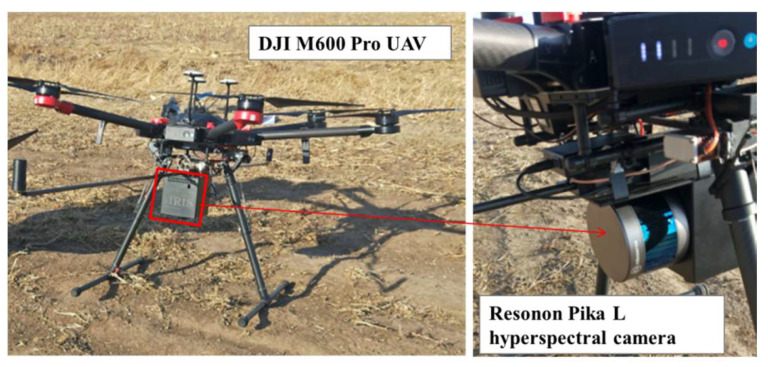
The UAV platform and the imaging hyperspectral sensor.

On the day when soil samples were collected, images were also obtained over the study area with Resonon Pika L hyperspectral camera loaded on the DJI M600 Pro UAV platform (Figure 3). The camera captured data in 281 spectral channels from 0.4 to 1.0 μm in the VIS-NIR region with a spectral resolution at 2.1 nm. In order to reduce noise and facilitate data transfer and fast processing, the spectral data were binned to 150 spectral bands each with 4 nm by the interpolation method of spline [37]. In this study, three impact factors were considered for setting up the flight altitude, including regional regulation and limitation of flight altitude, range of study area, and atmosphere influence. Therefore, flight height was set to 100 m, resulting in the spatial resolution of 0.1 m. Image overlap was set at 50% for the side (each parallel flight line). The obtained images were further mosaiced with SpectrononPro software (Spectronon Pro, Resonon Inc., Bozeman, MT, USA).

**Figure 4:**
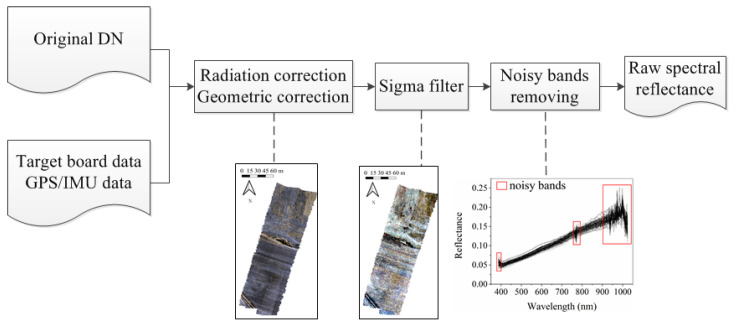
The flowchart of UAV data preprocessing.

As shown in Figure 4, a target board was applied for radiation correction and reflectance transformation from the Digital Number (DN) of the original image. Then, a geometric correction was performed on the recorded data with SpectrononPro, based on the onboard GPS and inertial measurement unit (IMU). The geometric correction accuracy was improved by base stations set up on the ground based on post-differential GPS processing [38]. It should be noted that the extremely high spatial resolution of UAV images may cause noises such as field monopoly shadows in the quantitative estimation of soil properties [19]. In our case, the spatial distance between two adjacent field monopoly was approximately 1 m, while the spatial resolution of the UAV data was 0.1 m. To eliminate these noises, the sigma filter method was conducted. Furthermore, a series of window sizes from 5 by 5 to 19 by 19 were tested for sigma filter. It was found that the sigma filter with the 11 by 11 window performed best with the highest correlation coefficient (r) value between spectra and SOM, STN. Finally, the noisy or troublesome spectral bands on the edge of spectra were removed. Consequently, 112 bands (403~759 nm and 783~900 nm) were retained, and then the raw spectral reflectance data were obtained.

### 2.3. Research Methods

#### 2.3.1. Spectral Denoising Methods

Spectral preprocessing can effectively remove spectral noise. One is scatter correction methods, including Multiplicative Scattering Correction (MSC) and Standard Normal Variate transformation (SNV). The other is spectra derivatives methods, including the first derivate (FD) and the second derivate (SD) [39].

MSC is effective in eliminating the scattering effect and enhancing the spectral absorption information related to the soil properties in the spectral data. The method first establishes an “ideal spectrum” of the samples, i.e., a direct linear relationship between the spectral variation and the content of soil nutrients in the samples. The established spectrum is used as the standard spectrum for all other sample spectra corrections, including baseline shift and offset corrections. Since the “ideal spectrum” is difficult to obtain, the average spectrum of all spectra is taken as the ideal one in practice. The specific algorithm works as follows: (i) calculate the average spectrum of all sample spectra; (ii) take the average spectrum as the ideal spectrum and perform linear regression to find the linear shift (regression constant $b_{i}$) and tilt offset (regression coefficient $a_{i}$) of each sample spectrum relative to the standard spectrum in Equation (1); (iii) subtract the linear shift from the original spectrum of each sample and divide it by the tilt offset, as in Equation (2).

(1) $$R_{ik}=b_{i}+a_{i}\bar{R_{k}}$$

where $R_{ik}$ denotes the reflectance of the i-th sample at the k-th band; $\bar{R_{k}}=\frac{\sum_{i=1}^{n}R_{ik}}{n}$, i=1,2,…,n, and n is the number of samples.

(2) $$R_{ik{\_}_{MSC}}=\frac{R_{ik}-b_{i}}{a_{i}}$$

SNV is applied to reduce the effects of particle size heterogeneity and surface nonspecific scattering. It is assumed that the reflectance of each spectral wavelength point meets specific distribution characteristics, such as normal distribution. Based on the assumption, each sample spectrum can be corrected by subtracting the average value of those spectra from the original sample spectrum and dividing by the standard deviation, which is calculated as follows:

(3) $$R_{ik{\_}_{SNV}}=\frac{R_{ik}-\bar{R_{i}}}{\sqrt{\frac{\sum_{k=1}^{m}{\left(R_{ik}-\bar{R_{i}}\right)}^{2}}{\left(m-1\right)}}}$$

where $\bar{R_{i}}=\frac{\sum_{k=1}^{m}R_{ik}}{m}$, k=1,2,…,m, and m is the number of bands.

FD and SD are the most common spectral derivatives methods. They can not only eliminate the baseline shifts and atmospheric scattering in the spectrum but also amplify the subtle changes in the slope of the spectral curve. These two methods are exploited to eliminate other background interference and improve discrimination and sensitivity. The spectral derivatives are expressed as follows:

(4) $$R_{ik{\_}_{FD}}=\frac{R_{i\left(k+1\right)}-R_{ik}}{\lambda_{k+1}-\lambda_{k}}$$

(5) $$R_{ik{\_}_{SD}}=\frac{R_{i\left(k+2\right)}-2R_{i\left(k+1\right)}+R_{ik}}{{\left(\lambda_{k+1}-\lambda_{k}\right)}^{2}}$$

where $\lambda_{k}$ denotes the wavelength of the *k*-th band.

#### 2.3.2. CARS-SPA Feature Selection

Competitive Adaptive Reweighted Sampling (CARS) is a sampling approach for feature band selection based on Monte Carlo sampling and PLSR [40]. Through supervised feature selection, a subset of bands sensitive to predictor variables can be selected, but there may still be redundant variables with high correlation across the bands. The Successive Projections Algorithm (SPA) is another feature selection algorithm that minimizes the collinearity in the vector space [41]. Based on CARS, SPA is further exploited to eliminate the collinearity between bands, which helps to make the selected feature bands contain the most information and the least internal similarity.

The main steps of the CARS algorithm are as follows: (i) use Monte Carlo sampling approach to select the set of calibration samples and establish the PLSR model; (ii) execute the enforced selection according to the exponentially decreasing function (EDF) based on the regression coefficient generated in each loop; (iii) competitively refine the remaining variables by the adaptive reweighted sampling (ARS); and (iv) evaluate each subset of selected spectral variables with cross validation [42]. The subset of variables with the lowest root mean square error of cross validation (RMSECV) is considered as the optimal set of feature bands [43].

The SPA algorithm is a forward variable selection algorithm based on vector projection analysis. The variables are selected from those with the largest projection value on the orthogonal subspace. First, we set N as the maximal number of variables to be selected. Then, the algorithm generates K sets of collections of N variables selected from the projected variable space. Finally, the set with the minimal RMSE of the multiple linear regression will be used to determine the optimal initial variable and the number of variables [44].

#### 2.3.3. Predicting Model and Evaluation of the Accuracy

PLSR is a linear modeling technique that projects information from the original independent variable space into a few latent variables, a.k.a., “PLSR components”. This simplifies the interpretation of the correlation between independent and dependent variables because PLSR uses the smallest possible component number. The spectra matrix is used as the independent variable, and the actual values of SOM and STN are used as the dependent variable. PLSR aims to recognize a model with an optimal number of components which yield the lowest RSME and the highest R^2^ values. A well fitted PLSR model indicates that the PLSR factors can provide explanation on the variation observed for predictors and responses [45]. In this way, the correlation between the actual values of SOM, STN, and the observed spectra matrix involves the optimal number of components [46]. This regression model was implemented in the R language with the version of 3.2.1 that is equipped with the package “pls” for PLSR modeling.

SVM is a kernel-based statistical machine learning method. As a popular data mining method, SVM has been used to model the VIS-NIR spectra data, and a nonlinear function is obtained by mapping a linear learning machine into a feature space induced by a high-dimensional kernel [47]. In this study, a radial basis function (RBF) kernel was selected, which can solve the non-linear problem using approximate multivariable function. The “e1071 package” with an R interface connected to the library for support vector machines (LIBSVM) was used. The optimization of the kernel-specific SVM parameters including C, e, and a, as well as the selection of the best preprocessing steps was performed by a systematic grid search using the leave-one-out cross-validation strategy applied on the training dataset.

**Figure 5:**
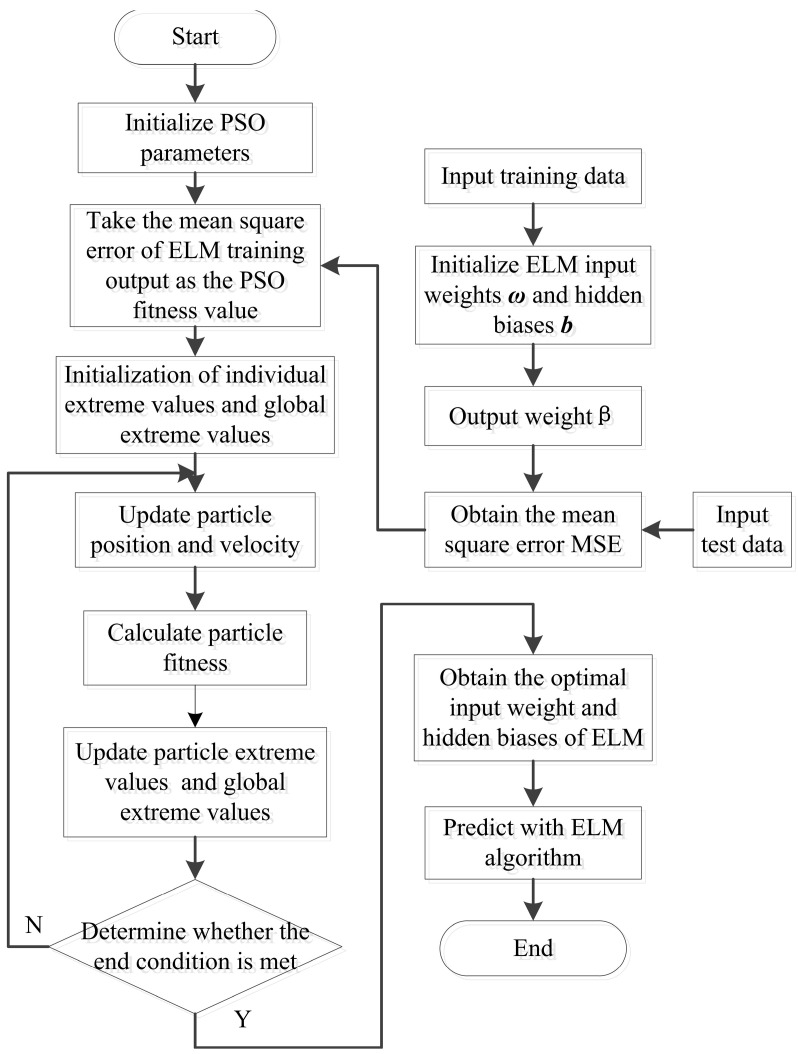
The flowchart of PSO-ELM.

Particle swarm optimization (PSO) is a computational method that simulates the foraging behavior of birds [48]. Exploiting PSO to optimize the selection of the input layer weights and hidden bias values of ELM can decrease the number of hidden layer nodes required by ELM and improve the generalization ability of a trained neural network. The process of ELM exploiting particle swarm optimization (PSO-ELM) is shown in Figure 5. There are four parameters that should be considered in the PSO method, including inertia weight, learning factors, maximum number of iterations, and population size. The parameter of inertia weight (w) has significance for performance of PSO. Shi and Eberhart [49] originally proposed adaptive inertia to assign optimal inertia weight. The strategy of decreasing weight of adaptive inertia was used in this study. The maximum value of w is chosen from the range of 0.8 to 1.2 for increasing the probability of locating the global optimum peak. The minimum value of w is chosen from the range of 0.4 to 0.2 to allow the particles to converge to the located optimum slowly [50]. Therefore, the inertia weights $w_{max}$ and $w_{min}$ were set to 0.8 and 0.4, respectively. The learning rates c1 and c2 are the acceleration constants with positive values [29], and c1 > 0, c2 ≤ 2 are respectively the individual (c1) and global acceleration (c2) coefficients. Further, c1 and c2 are set with the same values, but sometimes different values of c1 and c2 can help improve the model performance [51]. The appropriate values of c1 and c2 were obtained by performing a test with a step of 0.1. Finally, the values of c1 and c2 were respectively set to 2.4 and 1.6 for PSO, which present the best predictive parameters for this model. The maximum number of iterations T was set to 100. The population size was tested from 25 to 200 with a step of 25, and the population size of 100 with the highest prediction accuracy was used in the PSO. Then, the input weights and hidden biases corresponding to each particle were applied to the ELM model. Meanwhile, 55 training samples were used to train and 13 test samples were used for external validation. The mean square error (MSE) between the predicted and observed values of the test samples was used as the fitness of PSO to calculate the individual extreme value and global extreme value. The position and velocity of the particles were updated by particle fitness iteratively. The individual extremum and global extremum of the particles were updated until the minimum error was obtained or the maximum iteration number was reached. Finally, the input weights and hidden biases that yield the optimal results were used as the input parameters for the ELM model.

The coefficient of determination (R^2^) and the mean absolute percentage error (MAPE) were employed to validate the performance of the model. Meanwhile, the residual prediction deviation (RPD, the ratio between the standard deviation and the root mean square error) was used to assess the stability and accuracy of the multivariable models [52]. Existing studies generally assume that the models with RPD >2 are capable of providing accurate prediction of the discussed attributes; the models with RPD between 2 and 1.4 have moderate predictability, while the models with RPD <1.4 have poor predictive power [53].

## 3. Results

### 3.1. Analysis of Spectral Denoising Effect

**Figure 6:**
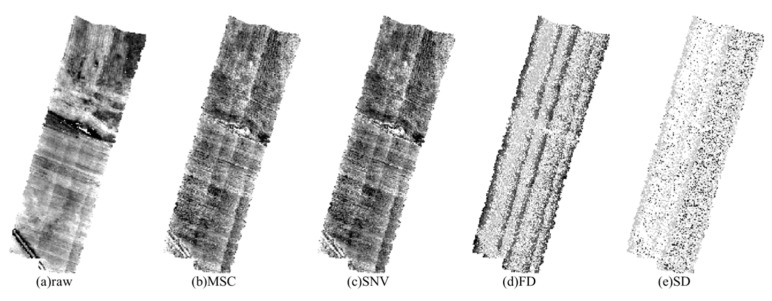
UAV hyperspectral images based on different spectral preprocessing.

The UAV images preprocessed by different methods are illustrated in Figure 6. By visual evaluation, the processed images by MSC and SNV can better present the typical features such as textures and smooth areas than those processed by FD and SD.

**Figure 7:**
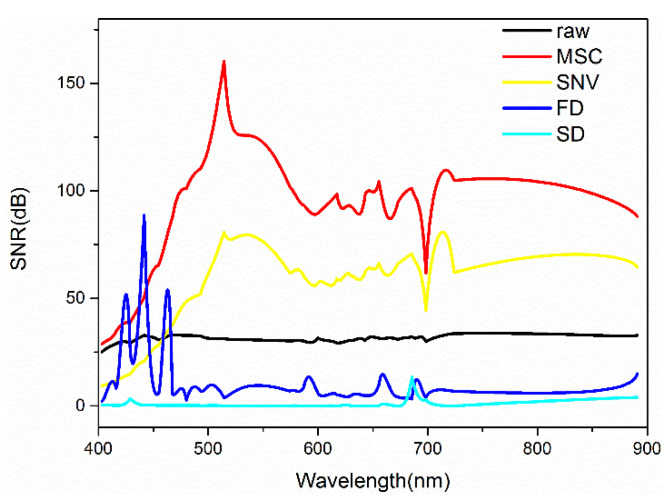
Signal-to-noise ratio of images based on different spectral preprocessing.

As shown in Figure 7, the signal-to-noise ratio (SNR) values of the images processed by MSC and SNV were higher than that of the raw image. Specifically, the MSC technique improved the SNR from 31 to 160 dB at 514 nm, and the SNV technique improved the SNR from 33 to 99 dB at 715 nm. Whereas, the FD and SD techniques generated images with much lower SNR.

**Figure 8:**
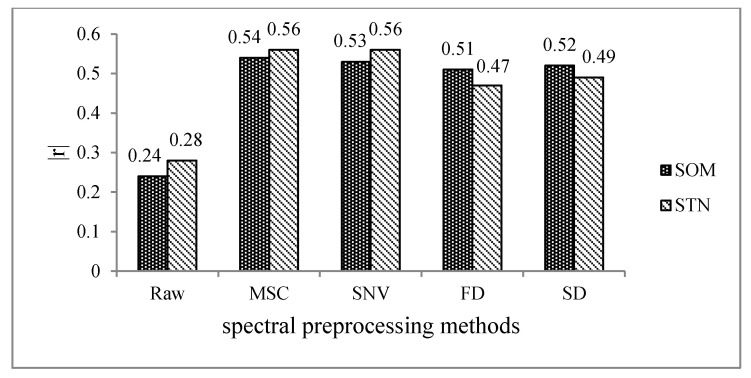
The absolute value of the maximum Pearson’s correlation coefficient (|r|) between sample spectra and soil properties based on different spectral preprocessing methods.

The correlation analysis of spectra with SOM and STN is illustrated in Figure 8. It can be seen that all four spectral preprocessing methods improved the correlation between spectra and soil properties. The MSC technique improved the correlation most, with the absolute value of the maximum correlation coefficient between spectral reflectance and soil properties being increased from 0.24 to 0.54 for SOM and from 0.28 to 0.56 for STN. Therefore, MSC can be regarded as the best spectral denoising method, and the subsequent feature selection and modeling were performed based on the spectral data processed by the MSC technique.

### 3.2. Spectral Feature Bands Selection

**Figure 9:**
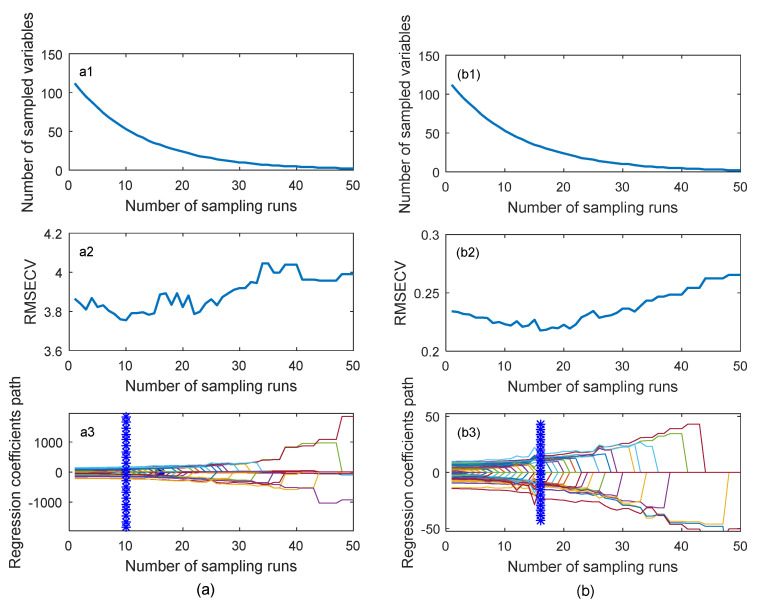
Screening spectral wavelength variables of SOM (**a**) and STN (**b**) based on CARS.

The feature selection process of 50 CARS runs for the 112 bands of the UAV hyperspectral image is shown in Figure 9, where the process of screening SOM and STN feature bands are exhibited in Figure 9(a1–a3) and Figure 9(b1–b3), respectively. It can be seen from Figure 9(a1,b1) that in the process of selecting spectral variables, the number of selected band variables gradually decreased with the increase of sampling runs, and the decreasing trend turned from fast to slow. The RMSECV curve in Figure 9(a2,b2) illustrates a trend of first decreasing to the lowest point and then increasing, indicating that the bands unrelated to SOM and STN were eliminated in the process first, and then the bands related to SOM and STN were eliminated. In Figure 9(a3,b3), the location of vertical star markers corresponded to the minimum RMSECV value in the entire variable screening process.

**Figure 10:**
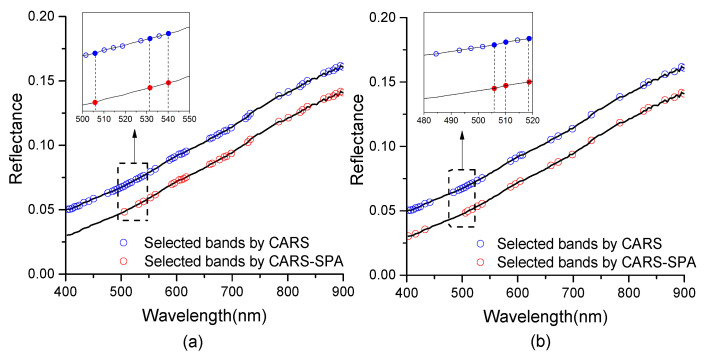
Feature bands of SOM (**a**) and STN (**b**) selected by CARS and CARS-SPA.

The number of SOM variables screened by CARS was 53, and the distribution of their band positions is shown in Figure 10a. It can be seen that the sensitive bands of SOM were located in the interval of 400~900 nm, but the distribution was relatively dense in 400~700 nm. The number of selected STN variables was 33, and the distribution of their band locations was shown in Figure 10b. It can be seen that the sensitive bands of STN were densely distributed in 400~440 nm and 480~540 nm. After the secondary screening of the feature bands through the SPA algorithm, the redundant bands at 400~540 nm were eliminated, and 24 SOM feature bands (Figure 10a) and 22 STN feature bands (Figure 10b) were finally obtained. It can be observed from the partial enlargement in the figures that in the range of 500~550 nm, 506, 532, and 540 nm were reserved by SPA for SOM prediction, and in the range of 480~520 nm, 506, 510, and 519 nm were reserved by SPA for STN prediction.

### 3.3. Model Accuracy

**Figure 11:**
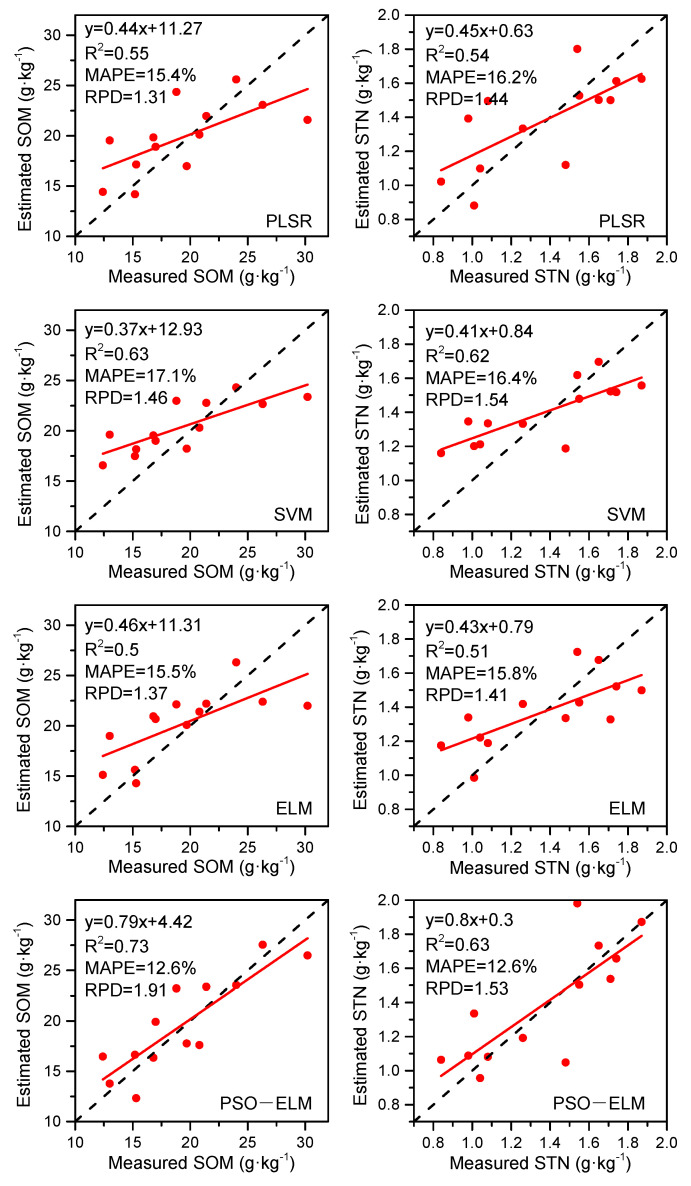
Scatter plots of the measured and estimated soil properties of test samples based on different models.

The PLSR, SVM, ELM, and PSO-ELM were conducted to predict SOM and STN based on the hyperspectral feature (Table 1 and Table 2). PLSR models based on full spectrum generated prediction accuracy for SOM estimation of R^2^ = 0.55, MAPE = 15.4%, and RPD = 1.31, for STN estimation of R^2^ = 0.54, MAPE = 16.2%, and RPD = 1.44. SVM, ELM, and PSO-ELM models with full spectrum as inputs achieved a poor performance with MAPE higher than 19% both for SOM and STN estimation. As PLSR model can select the hyperspectral feature based on “PLSR components”, thus, the CARS and CARS-SPA methods were not conducted in the PLSR model for input feature selection. Although the R^2^ values from SVM, ELM, and PSO-ELM were increased by using the CARS method to remove the irrelevant variables in the full spectrum, these models still have poor prediction ability with RPD value <1.4. Once the CARS-SPA method was exploited for secondary screening of feature bands, the accuracies of the SOM and STN prediction were further improved. The PSO-ELM model with selected bands by CARS-SPA produced the highest accuracy for SOM prediction with R^2^ = 0.73, MAPE = 12.6%, and RPD = 1.91 (Figure 11), for STN prediction with R^2^ = 0.63, MAPE = 12.6%, and RPD = 1.53 (Figure 11). Overall, the spectral variable selection method of CARS-SPA provided better estimates of SOM and STN than the full spectrum and CARS, which was indicated by the greater R^2^ and RPD values as well as lower MAPE values.

### 3.4. Soil Nutrient Spatial Distribution

**Figure 12:**
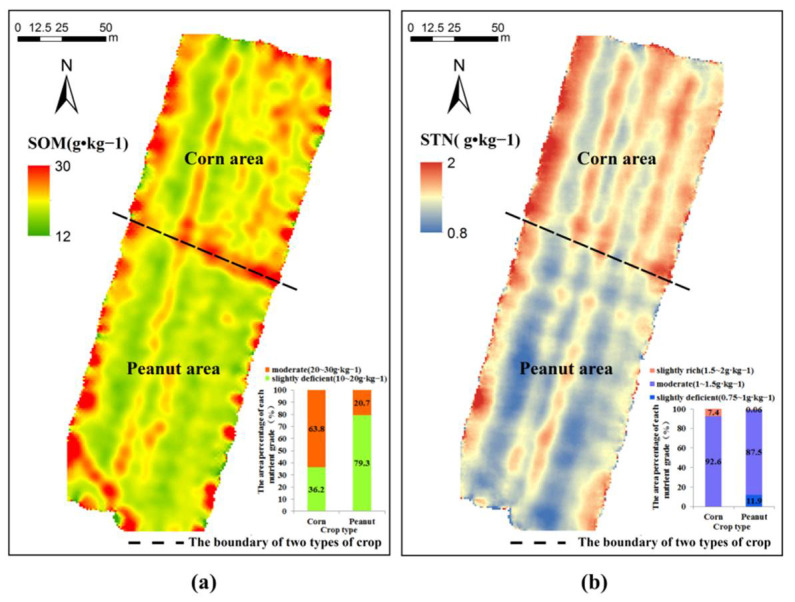
Spatial distribution of SOM (**a**) and STN (**b**) using UAV images based on optimal regression models.

The PSO-ELM model established based on the set of feature bands selected by the CARS-SPA algorithm was exploited to invert the SOM and STN pixel by pixel in the study area. The SOM content was between 12 and 30 g kg^−1^, and the STN content was between 0.8 and 2 g kg^−1^. The spatial distribution of SOM and STN was shown in Figure 12. It can be seen that the high values of SOM and STN were mainly located in the northeastern corn growing area. According to the nutrient grading standard of agricultural soils, the area percentage of each nutrient grade in corn and peanut areas was counted separately, and the result is shown in the lower right corner of the figure. The analysis indicated that the amount of SOM in the corn area was moderate while that in the peanut area was slightly deficient; the amount of STN in both the corn and peanut areas was moderate.

## 4. Discussion

Preprocessing of hyperspectral imagery can facilitate subsequent spectral interpretation and data modeling since unwanted noise can be eliminated while chemical signals of interest can be enhanced [54]. Meanwhile, the development of spectroscopic equipment has brought new challenges for preprocessing spectral data. Preprocessing is exploited to standardize spectra and remove instrumental and physical noise. In this study, the preprocessing based on the MSC, SNV, FD, and SD techniques was considered before spectral feature selection and modeling. It is essential to reduce additive and multiplicative effects caused by light scattering to achieve an accurate quantitative analysis of soil properties on the hyperspectral data obtained from UAV platforms. The SNR of raw imagery without any preprocessing was approximately 30 dB, and a weak correlation was found between the spectral characteristic and soil properties. To achieve optimal prediction performances, it was recommended that powerful preprocessing techniques such as MSC and SNV should be given priority so as to increase the SNR value and the correlation coefficient with soil properties. The MSC technique is based on the Beer–Lambert model, which can reduce the wavenumber independent radiation loss caused by scattering or stray light [55]. The SNV technique has the advantage of handling a constant offset. To remove the baseline offset or handle the baseline polynomial fitting for UAV imagery, MSC is recommended as a better practice than SNV. It was also found that MSC performed better in soil clay estimation on CHRIS images [53]. FD and SD are two common and popular techniques to resolve overlapping analyte signals through conversion of spectra [56]. Since the peak position of spectra is crucial for indicating chemical properties, the first or second derivatives transformation can reserve or highlight band positions [55]. However, there is no obvious peak position or absorption feature for soil spectra in 400~100 nm from UAV hyperspectral imagery. This may make the FD or SD method not perform well to reduce or eliminate the scatter effect in the preprocessing of imagery.

To reduce the large dimensionality of the hyperspectral image data and to decrease the computation time, optimal wavelength selection may be more significant than using a full spectrum for the quantitative model [57]. The SVM, ELM, and PSO-ELM models established based on full spectrum obtained the RPD less than 1.4 for SOM and STN, indicating no prediction ability. Additionally, the overlarge number of variables and a small number of samples may cause an overfitting problem in the prediction model [58]. In this study, the input bands that produce high prediction accuracy with minimum collinearity are considered as the optimal wavelengths. CARS based on the backward stepwise method can fast select variables to obtain high prediction accuracy [59]. Exploiting this approach, the effective primary wavelengths were selected from 112 bands of hyperspectral imagery, i.e., 53 bands for SOM and 33 bands for STN, which reduced the MAPE value from 24.9% to 18.6% for SOM and from 19.2% to 15.2% for STN using PSO-ELM. Furthermore, to reduce the collinearity effects from primary wavelengths, the SPA algorithm was exploited for a secondary wavelength selection, which obtained 24 bands for SOM and 22 bands for STN with small collinearity. The experimental results revealed that in contrast to selecting wavelengths only by primary spectral, the coupling of CARS with SPA could reduce the number of bands and improve the prediction accuracy.

Hyperspectral imagery can be considered as multidimensional big data, such as a matrix of 2820 × 1500 pixels multiplied with 112 bands in our study area covering 0.043 km^2^. A sound and effective learning algorithm is required for soil prediction and mapping on hyperspectral imagery at various scales. The ELM method with random weights of the hidden neurons and inherent steps has benefits in high training speed and easily ensemble [60]. However, ELM usually requires more hidden neurons than traditional algorithms to make good predictions, which might result in slow responses of ELM to new data [26]. To handle this problem, PSO was employed to optimize the input weights and hidden biases, which can improve the generalization performance of ELM with fewer hidden neurons. In this paper, a comparison of the PSO-ELM and ELM regression models was conducted. The greater RPD and R^2^ values as well as lower MAPE values indicated that the PSO-ELM methods provided better estimation than the ELM methods for both SOM and STN. It was also found that in the forecasting of CO_2_ emissions, the established PSO-ELM model outperformed the ELM [61]. The results of this paper also showed that PSO-ELM performed better than PLSR and SVM with the greater R^2^ and lower MAPE values.

## 5. Conclusions

The experimental results in this study revealed that UAV spectroscopy imaging can be exploited to quantify the SOM and STN in farmland. This study explored the wavelengths selection of UAV hyperspectral imagery and an improved extreme learning machine method for SOM and STN prediction and mapping. The CARS coupled with SPA was a potential strategy for identifying optimal and effective bands for hyperspectral imagery. The following conclusions were made:

1. This work proved that the preprocessing to reduce unwanted noise for UAV imagery is essential to establish optimal models for predicting SOM and STN with VIS-NIR spectroscopy. The MSC technique was highly recommended for preprocessing, which contributed to the image radiance with high SNR value and reflectance spectra with high correlation coefficient.
2. The CARS-SPA approach could select a small number of reasonable wavelengths, which selected 33 and 22 bands that were informative for SOM and STN prediction. Based on these bands, the prediction performance was exhibited by R^2^ of 0.73, MAPE of 12.6%, and RPD of 1.91 for SOM and R^2^ of 0.63, MAPE of 12.6%, and RPD of 1.53 for STN with the PSO-ELM model.
3. The PSO was exploited to assign the weights and biases of the ELM model while avoiding randomness. The proposed method of PSO-ELM outperformed the ELM in that it reduced the MAPE by 2.9% and 3.2% and increased R^2^ by 0.23 and 0.12 for SOM and STN, respectively. It also performed better than PLSR and SVM with the greater R^2^ and lower MAPE values.

Overall, this study provided an alternative approach for soil properties estimation and mapping based on the imaging spectrometry obtained from a UAV platform. In addition, utilizing imagery and field samples on a larger scale will be our next-step research toward developing regional models.

## Figures and Tables

**Table 1:** Model accuracies of test samples based on different feature selection methods for predicting SOM.

Modeling Method	Feature Selection Method	R^2^	MAPE	RPD
PLSR	Full spectrum	0.55	15.4%	1.31
SVM	Full spectrum	0.18	20.2%	1.14
	CARS	0.35	18.8%	1.26
	CARS-SPA	0.63	17.1%	1.46
ELM	Full spectrum	0.24	24.3%	0.99
	CARS	0.35	22.5%	1.00
	CARS-SPA	0.50	15.5%	1.37
PSO-ELM	Full spectrum	0.26	24.9%	1.05
	CARS	0.55	18.6%	1.42
	CARS-SPA	0.73	12.6%	1.91

**Table 2:** Model accuracies of test samples based on different feature selection methods for predicting STN.

Modeling Method	Feature Selection Method	R^2^	MAPE	RPD
PLSR	Full spectrum	0.54	16.2%	1.44
SVM	Full spectrum	0.34	19.1%	1.26
	CARS	0.57	16.9%	1.46
	CARS-SPA	0.62	16.4%	1.54
ELM	Full spectrum	0.26	22.0%	1.02
	CARS	0.43	16.5%	1.28
	CARS-SPA	0.51	15.8%	1.41
PSO-ELM	Full spectrum	0.27	19.2%	1.05
	CARS	0.58	15.2%	1.41
	CARS-SPA	0.63	12.6%	1.53
